# Does the methanogenesis inhibitor 3-nitrooxypropanol affect milk yield in dairy cattle?

**DOI:** 10.18849/ve.v10i4.717

**Published:** 2025-11-25

**Authors:** Justine Cole+

**Keywords:** 3-NITROOXYPROPANOL, DAIRY COWS, ENVIRONMENTAL TECHNOLOGY, FEED ADDITIVE, MILK YIELD, NUTRITION

## Abstract

**PICO question:**

In dairy cattle, does the methanogenesis inhibitor 3-nitrooxypropanol affect milk yield, compared to cows that receive no intervention to reduce enteric methane emissions?

**Clinical bottom line:**

**Category of research:**

Treatment.

**Number and type of study designs reviewed:**

A total of 15 controlled trials were critically reviewed, of which 11 were randomised.

**Strength of evidence:**

Moderate.

**Outcomes reported:**

Eleven studies reported no effect of 3-nitrooxypropanol (3-NOP) on milk yield at doses ranging from 40 to 135 mg of 3-nitrooxypropanol/kg of feed dry matter (DM). Two studies reported trends for decreased milk yield at doses greater or equal to 80 mg of 3-NOP/kg of feed DM, while a further two studies reported statistically significant declines in milk yield at doses of 80 mg of 3-NOP/kg of feed DM.

**Conclusion:**

The reviewed studies provide a moderate strength of evidence to support that the administration of the methanogenesis inhibitor 3-NOP at concentrations less than 80 mg/kg of feed DM does not significantly affect milk yield in dairy cattle. Some studies suggest that 3-NOP may adversely affect milk yield at higher doses but the results are inconsistent.

## How to apply this evidence in practice

The application of evidence into practice should take into account multiple factors, not limited to: individual clinical expertise, patient’s circumstances and owners’ values, country, location or clinic where you work, the individual case in front of you, the availability of therapies and resources.

Knowledge Summaries are a resource to help reinforce or inform decision-making. They do not override the responsibility or judgement of the practitioner to do what is best for the animal in their care.

## Clinical scenario

A client with a dairy farm has recently observed a decrease in milk yield in some of their cows. The only management change that has occurred since your last routine visit is the addition of a feed additive that reduces enteric methane emissions. The product, 3-nitrooxypropanol (3-NOP), is administered via the herd’s mixed rations on a continuous basis at 60 mg of 3-NOP/kg of feed dry matter (DM) and has been effective in reducing the herd’s methane emissions. The farmer is concerned, however, that this product might be reducing the milk yield of their herd and has asked you if this is possible. You want to learn more about this additive and its effects on milk yield before further advising the farmer on this product.

## The evidence

Research to date has primarily focused on the efficacy of the compound in reducing methane emissions (Bampidis et al., 2021), rather than the effects 3-NOP may have on milk production. The fifteen appraised studies presented here should be interpreted with caution as they have several limitations. Firstly, variations in study design, basal diet composition, dosage of 3-NOP, and lactation stage of the cows preclude direct comparison of the studies. Secondly, most of the critically reviewed studies were limited by lack of blinding or power analyses and evaluation of low doses of 3-NOP. Finally, several of the studies were further limited by the lack of a covariate period for baseline data collection, washout and/or experimental periods of insufficient duration, failure to quantify the dose of 3-NOP in the experimental diets, or their approach to the removal of subjects and subsequent missing data.

Eleven studies (Haisan et al., 2014; 2017; Hristov et al., 2015; Lopes et al., 2016; Melgar et al., 2020a; 2021; Van Gastelen et al., 2020; 2024; Schilde et al., 2021; Van Wesemael et al., 2019; Ma et al., 2024) reported no effect of 3-NOP on milk yield at doses ranging from 40 to 135 mg of 3-nitrooxypropanol/kg of feed dry matter (DM). Two studies reported trends for decreased milk yield at 3-NOP doses greater or equal to 80 mg of 3-nitrooxypropanol/kg of feed DM (Melgar et al., 2020b; Kjeldsen et al., 2024), while a further two studies reported statistically significant declines in milk yield at 3-NOP doses of 80 mg of 3-nitrooxypropanol/kg of feed DM (Van Gastelen et al., 2022; Maigaard et al., 2024). The reviewed studies support that 3-nitrooxypropanol concentrations up to 80 mg of 3-NOP/kg of feed DM do not significantly affect milk yield in dairy cattle. There is, however, limited evidence to suggest that 3-NOP may adversely affect milk yield at higher doses. Further investigation of the relationship between 3-NOP concentration and milk yield is therefore necessary, as it is likely to inform regulatory decisions and influence the adoption of this feed additive by the dairy industry.

## Summary of the evidence

**Haisan et al. (2014):** The effects of feeding 3-nitrooxypropanol on methane emissions and productivity of Holstein cows in mid lactation **Aim: **To determine the effects of 3-nitrooxypropanol (3-NOP) on methane emissions, rumen fermentation, and milk production in lactating Holstein cows.

Population:	Primiparous and multiparous Holstein cows (Canada).
Sample size:	12 cows.
Intervention details:	Cows were split into two groups based on their pre-experimental days in milk (DIM). Group 1: n = 8 (4 primiparous and 4 multiparous), DIM = 100 ± 5.4. Group 2: n = 4 (2 primiparous and 2 multiparous), DIM: 76 ± 10.1. All cows in the experiment were fitted with ruminal cannulas. Cows received 2 dietary treatments in a cross-over experimental design. Control*:* silicon dioxide (SiO_2_) only. 130NOP*:* 5 mg of 3-NOP/kg of feed dry matter (administered as 2,500 mg of 3-NOP/cow/day) in a carrier powder of SiO_2_. The experiment ran for 56 days total and consisted of two periods of 28 days each. Each period consisted of an adaptation period of 21 days and a data collection period of 7 days. The study protocol was conducted in two groups, staggered by 7 days. Cows were provided with a total mixed ration (TMR) of feed at approximately 09:00, allowing for continuous intake throughout the day. The treatment and control compounds were manually mixed into the TMR within 30 minutes of feeding.
Study design:	Controlled trial.
Outcome Studied:	Data was collected on rumen gas emissions, milk production, feed consumption, rumen fluid composition, milk composition, and body weight.
Main Findings (relevant to PICO question):	No significant difference in individual cows' milk yields were detected between the treatment and control periods (P = 0.30). Other outcomes studied that are not relevant to the PICO question will not be discussed further.
Limitations:	No power calculation is reported, so it is unclear whether the limited sample size was adequate to detect differences between treatment and control periods. The study exhibits selection bias towards a higher producing dairy breed (Holsteins only). The study investigated the effects of 3-NOP on cows past the peak lactation period (> 4–8 weeks postpartum). The 3-NOP and the control SiO2 powders were both hand mixed into the cows' daily rations, increasing the possibility of uneven distribution of 3-NOP. The study failed to quantify the concentration of 3-NOP in the refusals of the TMR, thus the exact dosage of 3-NOP that cows received cannot be confirmed. Possible carryover effects cannot be excluded due to cross-over experimental design and current lack of knowledge on adequate washout periods for 3-NOP. The experimental period was too short in duration (< 28 days) to address whether repeated or long-term use of 3-NOP may result in adaptation by the rumen microbiota. One of the authors was stated as an employee of dsm-firmenich, manufacturer of Bovaer^®^, the tradename for 3-NOP.

**Haisan et al. (2017):** The effects of feeding 3-nitrooxypropanol at two doses on milk production, rumen fermentation, plasma metabolites, nutrient digestibility, and methane emissions in lactating Holstein cows **Aim: **To determine the effects of feeding 3-nitrooxypropanol (3-NOP) in the total mixed ration (TMR) on rumen fermentation, microbial populations, methane production, milk production, nutrient digestibility, and blood metabolites of lactating Holstein cows.

Population:	Primiparous and multiparous Holstein cows (Canada).
Sample size:	15 cows.
Intervention details:	The experiment was conducted using three groups of cows: Group 1: n = 6 primiparous cows in squares 1 and 2 Group 2: n = 6 multiparous cows in squares 3 and 4 Group 3: n = 3 multiparous cows in square 5. All cows in the experiment were fitted with ruminal cannulas. Cows were allocated to a Latin square and received three dietary treatments: Control: silicon dioxide (SiO_2_) only (administered as 2,500 mg/cow/day) 70NOP: 68.3 mg of 3-NOP /kg of feed dry matter and SiO_2_ (administered as 1,250mg of 3-NOP/cow/day + 1,250 of SiO_2_ mg/cow/day) 130NOP: 132.3 mg of 3-NOP /kg of feed dry matter (administered as 2,500 mg of 3-NOP/cow/day) in a carrier powder of SiO_2_. The experiment ran for 84 days total and consisted of three periods of 28 days each. Each period consisted of an adaption period of 20 days and a data collection period of 8 days. Cows were provided a total mixed ration (TMR) of feed at approximately 09:00, allowing for continuous intake throughout the day. The treatment and control compounds were mixed into the TMR manually.
Study design:	Controlled trial.
Outcome Studied:	Data was collected on rumen gas emissions, milk production, feed consumption, apparent total tract digestibility of nutrients (%), rumen fluid composition, milk composition, body weight, blood chemistry, and hormones.
Main Findings (relevant to PICO question):	Milk yield did not differ significantly between the control and treatment periods at 70NOP or 130NOP (P = 0.32). The data from one cow was removed from the experiment due to unknown illness and an extremely low DMI, resulting in data for only 14 cows being included for the lower 3-NOP dose. Other outcomes studied that are not relevant to the PICO question will not be discussed further.
Limitations:	No power calculation is reported, so it is unclear whether the sample size was adequate to detect differences between treatment and control periods. The experiment was non-blinded. The study exhibits selection bias towards a higher producing dairy breed (Holsteins only). The study investigated the effects of 3-NOP on cows past the peak lactation period (> 4–8 weeks postpartum). The 3-NOP and the control SiO2 powders were both hand mixed into the cows' daily rations, increasing the possibility of uneven distribution of 3-NOP. The study failed to quantify the concentration of 3-NOP in the refusals of the TMR, so the exact dosage of 3-NOP that cows received cannot be confirmed. Possible carryover effects cannot be excluded due to Latin square experimental design and current lack of knowledge on adequate washout periods for 3-NOP. The experimental period was too short in duration (< 28 days) to address whether repeated or long-term use of 3-NOP may result in adaptation by the rumen microbiota. One of the authors was stated as an employee of dsm-firmenich, manufacturer of Bovaer®, the tradename for 3-NOP. The authors also acknowledge the financial support of dsm-firmenich for this study.

**Hristov et al. (2015):** An inhibitor persistently decreased enteric methane emission from dairy cows with no negative effect on milk production **Aim: **To determine the effect of 3-nitrooxypropanol (3-NOP) on enteric methane emission in lactating Holstein cows.

Population:	Primiparous and multiparous Holstein cows (Pennsylvania State University Dairy Center, USA).
Sample size:	48 cows.
Intervention details:	Cows were blocked by DIM, parity, and milk yield into 12 blocks, consisting of 4 cows each. Cows from each block were randomly allocated to one of four treatment groups: Control: silicon dioxide (SiO_2_) and propylene glycol (1,2 propanediol) only (n = 12) 40NOP: 40 mg of 3-NOP/kg of feed dry matter, mixed with a carrier of SiO_2_ and propylene glycol (1,2 propanediol) (n = 12) 60NOP: 60 mg of 3-NOP/kg of feed dry matter, mixed with a carrier of SiO_2_ and propylene glycol (1,2 propanediol) (n = 12) 80NOP: 80 mg of 3-NOP/kg of feed dry matter, mixed with a carrier of SiO_2_ and propylene glycol (1,2 propanediol) (n = 12). The experiment was conducted in two phases, each consisting of a covariate period lasting 2 weeks and an experimental period lasting 12 weeks. Cows were provided a total mixed ration (TMR) of feed allowing for continuous intake throughout the day. The treatment and control mixtures were mixed into TMR (specific details not reported).
Study design:	Randomised controlled trial.
Outcome Studied:	Data was collected on rumen gas emissions, milk production, feed consumption, apparent total tract digestibility of nutrients (%), milk composition, and body weight.
Main Findings (relevant to PICO question):	No statistically significant effects were observed for milk yield: control vs. all 3-NOP treatments (P = 0.59) linear effect of 3-NOP (P = 0.21) quadratic effect of 3-NOP (P = 0.19). Other outcomes studied that are not relevant to the PICO question will not be discussed further.
Limitations:	No power calculation was reported for the experiment. The experiment was non-blinded. The study exhibits selection bias towards a higher producing dairy breed (Holsteins only). The study investigated the effects of 3-NOP on cows past the peak lactation period (> 4–8 weeks postpartum). The study investigated relatively low doses of the compound (40, 60, 80 mg of 3-NOP /kg of feed dry matter) as all doses were less than the maximum concentration of 100 mg of 3-NOP/kg of feed dry matter approved by the European Food Safety Authority. The experimental period was too short in duration (< 28 days) to address whether repeated or long-term use of 3-NOP may result in adaptation by the rumen microbiota. No specific details on how 3-NOP was mixed into the TMR were included in their experimental methods, increasing the possibility of uneven distribution of 3-NOP throughout the TMR. The study failed to quantify the concentration of 3-NOP in the refusals of the TMR, thus the exact dosage of 3-NOP that cows received cannot be confirmed. Two of the authors are listed as employees of dsm-firmenich, manufacturer of Bovaer^®^, the tradename for 3-NOP.

**Kjeldsen et al. (2024):** Gas exchange, rumen hydrogen sinks, and nutrient digestibility and metabolism in lactating dairy cows fed 3-nitrooxypropanol and cracked rapeseed **Aim: **To determine the effects of cracked rapeseed and 3-nitrooxypropanol (3-NOP) on gas exchange, dry matter intake (DMI), nutrient digestion, and nutrient metabolism in Holstein dairy cows.

Population:	Multiparous Holstein cows (Cattle Research Centre, Aarhus University, Denmark).
Sample size:	4 cows.
Intervention details:	All cows were multi-cannulated (rumen cannula and simple T-cannulas in the duodenum and ileum). Dietary treatments were organised in a 2 × 2 factorial arrangement with 2 supplementation levels for dietary fat and 3-nitrooxypropanol (3-NOP). The low fat (LF) treatment was 33 g of crude fat/kg of dry matter and the high fat (HF) treatment was 64 g of crude fat/kg of dry matter The control treatment consisted of only silicon dioxide (SiO_2_) and propylene glycol (1,2 propanediol), while the 80NOP treatment consisted of 80 mg of 3-NOP/kg of feed dry matter, mixed with a carrier of SiO_2_ and propylene glycol (1,2 propanediol). Cows were then randomly allocated to a Latin square and received four dietary treatments: LF x Control HF x Control LF x 80NOP HF x 80NOP. The experiment ran for 84 days total and consisted of 4 periods of 21 days each. Each period consisted of an adaptation period of 11 days, a 5-day period for ruminal sampling and a 5-day period for gas data collection. Cows were provided a total mixed ration (TMR) of feed at 06:15 and 17:10, allowing for continuous intake throughout the day. The SiO_2_ and 3-NOP were mixed into the TMR by an auger mixer. The LF and HF control diets containing the placebo were mixed first, followed by a cleaning procedure, and then the LF and HF 3-NOP diets were mixed.
Study design:	Controlled trial.
Outcome Studied:	Data was collected on rumen gas emissions, milk production, feed consumption, apparent and true total tract digestibility of nutrients (%), nutrient intake, rumen digesta and fluid composition, milk composition, body weight, and blood metabolites.
Main Findings (relevant to PICO question):	A tendency for cows fed 3-NOP to have decreased milk yield (7.2%) was observed (P = 0.06). Other outcomes studied that are not relevant to the PICO question will not be discussed further.
Limitations:	No power calculation reported, despite an extremely limited sample size (n = 4). The experiment was non-blinded. The study exhibits selection bias towards a higher producing dairy breed (Holsteins only). There is also a selection bias towards higher producing multiparous cows, as primiparous cows were not included in this study population. Possible carryover effects cannot be excluded due to Latin squares experimental design and current lack of knowledge on adequate washout periods for 3-NOP. The study only investigated a single low dose of the compound (80 mg of 3-NOP/kg of feed dry matter), as 60 mg of 3-NOP/kg of feed dry matter is the minimum dose approved by the European Food Safety Authority. The experimental period was too short in duration (< 28 days) to address whether repeated or long-term use of 3-NOP may result in adaptation by the rumen microbiota. Two of the authors are listed as employees of dsm-firmenich, manufacturer of Bovaer®, the tradename for 3-NOP.

**Lopes et al. (2016):** Effect of 3-nitrooxypropanol on methane and hydrogen emissions, methane isotopic signature, and ruminal fermentation in dairy cows **Aim: **To determine the effect of 3-nitrooxypropanol (3-NOP) on enteric methane emission, ruminal microbial profile, and production variables in lactating dairy cows.

Population:	Primiparous and multiparous Holstein cows (Pennsylvania State University Dairy Center, USA).
Sample size:	6 cows.
Intervention details:	Cows were grouped by DIM and milk yield into two groups, consisting of 3 cows each. All cows in the experiment were fitted with ruminal cannulas. Cows received two dietary treatments in a cross-over experimental design: Control: silicon dioxide (SiO_2_) and propylene glycol (1,2 propanediol) only 60NOP: 60 mg of 3-NOP/kg of feed dry matter, mixed with a carrier of SiO_2_ and propylene glycol (1,2 propanediol). All cows received recombinant bovine somatotropin (bST) on the first day of each experimental period. The experiment ran for 28 days total and consisted of two periods of 14 days each. Each period consisted of an adaptation period of 10 days and a data collection period of 4 days. A washout period of 7 days was also implemented between experimental periods. Cows were provided with a total mixed ration (TMR) once daily at approximately 08:00, allowing for continuous intake throughout the day. The treatment and control compounds were mixed into TMR (specific details not reported).
Study design:	Controlled trial.
Outcome Studied:	Data was collected on rumen gas emissions, milk production, rumen fluid composition, feed consumption, and milk composition.
Main Findings (relevant to PICO question):	No significant difference in milk yield was detected between the treatment and control periods (P = 0.37). Data for one cow (control) was removed due to low milk production during period 2. Other outcomes studied that are not relevant to the PICO question will not be discussed further.
Limitations:	No power calculation is reported, so it is unclear whether the limited sample size was adequate to detect differences between treatment and control periods. The experiment was non-blinded. The study exhibits selection bias towards a higher producing dairy breed (Holsteins only). The number of multiparous vs. primiparous cows is not reported. The study investigated the effects of 3-NOP on cows past the peak lactation period (> 4–8 weeks postpartum). The study only investigated a single low dose of the compound, as 60 mg of 3-NOP/kg of feed dry matter is the minimum dose approved by the European Food Safety Authority. Administration of recombinant bovine somatotropin (bST) acted as a confounding variable as it may have increased the milk yield of cows included in the study. bST was also not included as a fixed variable in the model, and therefore an interaction between bST and 3-NOP could not be accounted for. bST is not approved for use in cattle in the UK or EU, which may limit the relevance of the results to UK dairy producers. No specific details on how 3-NOP was mixed into the TMR were included in their experimental methods, increasing the possibility of uneven distribution of 3-NOP throughout the TMR. The study failed to quantify the concentration of 3-NOP in the refusals of the TMR, so the exact dosage of 3-NOP that cows received cannot be confirmed. Possible carryover effects cannot be excluded due to cross-over experimental design and current lack of knowledge on adequate washout periods for 3-NOP. The experimental period was too short in duration (< 28 days) to address whether repeated or long-term use of 3-NOP may result in adaptation by the rumen microbiota. Two of the authors are listed as employees of dsm-firmenich, manufacturer of Bovaer®, the tradename for 3-NOP.

**Ma et al. (2024):** Effects of 3-nitrooxypropanol (Bovaer10) and whole cottonseed on milk production and enteric methane emissions from dairy cows under Swiss management conditions **Aim: **To determine the potential effect of 3-nitrooxypropanol (3-NOP) and whole cottonseed (WCS) on lactational performance and enteric methane emission of Holstein dairy cows.

Population:	Multiparous Holstein-Friesian and Brown Swiss cows housed in a free stall barn (ETH Zürich, Agrovet-Strickhof Research Station, Switzerland).
Sample size:	16 cows.
Intervention details:	Dietary treatments were organised in a 2 × 2 factorial arrangement with 2 supplementation levels for whole cotton seed (WCS) and 3-nitrooxypropanol (3-NOP). The basal total mixed ration (BTMR) treatment contained no whole cottonseed, while the whole cottonseed (WCS) treatment was supplemented with 50 g of whole cottonseed/kg of feed dry matter. The control treatment consisted of only silicon dioxide (SiO_2_) and propylene glycol (1,2 propanediol), while the 60NOP treatment consisted of 60 mg of 3-NOP/kg of feed dry matter, mixed with a carrier of SiO_2_ and propylene glycol (1,2 propanediol). The cows were arranged in a split-plot design based on breed, with one subplot containing 8 Holstein-Friesian cows and the other containing 8 Brown Swiss cows. All cows within each subplot were then randomly allocated to a Latin square and received 4 dietary treatments: BTMR x Control BTMR x 60NOP WCS x Control WCS x 60NOP. The experiment ran for 96 days total and consisted of four periods of 24 days each. Each period consisted of an adaptation period of 19 days and a data collection period of 5 days. Cows were provided a total mixed ration (TMR) of feed twice daily at 08:00 and 18:00 allowing for continuous intake throughout the day. The treatment and control compounds were mixed into TMR (specific details not reported).
Study design:	Randomised controlled trial.
Outcome Studied:	Data was collected on rumen gas emissions, milk production, feed consumption, apparent total tract digestibility of nutrients (%), nutrient intake, milk composition, and body weight.
Main Findings (relevant to PICO question):	No significant difference in milk yield was detected between the treatment and control periods (P = 0.91). Other outcomes studied that are not relevant to the PICO question will not be discussed further.
Limitations:	No power calculation reported, so it is unclear whether the limited sample size was adequate to detect differences between treatment and periods. There was a relatively small number of cows (n = 8) per breed. The experiment was non-blinded. The study exhibits a selection bias towards higher producing multiparous cows, as primiparous cows were not included in this study population. The study investigated the effects of 3-NOP on cows past the peak lactation period (> 4–8 weeks postpartum). Possible carryover effects cannot be excluded due to Latin squares experimental design and current lack of knowledge on adequate washout periods for 3-NOP. The study only investigated a single low dose of the compound, as 60 mg of 3-NOP/kg of feed dry matter is the minimum dose approved by the European Food Safety Authority. The experimental period was too short in duration (< 28 days) to address whether repeated or long-term use of 3-NOP may result in adaptation by the rumen microbiota. The study failed to quantify the concentration of 3-NOP in the refusals of the TMR, so the exact dosage of 3-NOP that cows received cannot be confirmed. No specific details on how 3-NOP was mixed into the total mixed ration (TMR) were included in their experimental methods, increasing the possibility of uneven distribution of 3-NOP throughout the TMR.

**Maigaard et al. (2024):** Effects of dietary fat, nitrate, and 3-nitrooxypropanol and their combinations on methane emission, feed intake, and milk production in dairy cows **Aim: **To determine the effect of dietary fat, nitrate, and 3-nitrooxypropanol (3-NOP) on dairy cows' enteric methane emissions and production performance.

Population:	Primiparous and multiparous Holstein cows in a loose housing system (AU Viborg-Research Centre Foulum of Aarhus University, Denmark).
Sample size:	48 cows.
Intervention details:	Cows were housed in two pens according to parity (primiparous or multiparous) and were further blocked by parity and days in milk (DIM) into 6 blocks, consisting of 8 cows each. Dietary treatments were organised in a 2 × 2 × 2 factorial arrangement with 2 supplementation levels for dietary fat, dietary nitrate, and 3-nitrooxypropanol (3-NOP) The low fat (LF) treatment was 30 g of crude fat/kg of dry matter and the high fat (HF) treatment was 63 g of crude fat/kg of dry matter. The nitrate treatment was 10 g of nitrate/kg of dry matter, while the urea treatment contained 0 g of nitrate/kg of dry matter but included urea as an alternative nitrogen source. The control treatment consisted of only silicon dioxide (SiO_2_) and propylene glycol (1,2 propanediol), while the 80NOP treatment consisted of 80 mg of 3-NOP/kg of feed dry matter, mixed with a carrier of SiO_2_ and propylene glycol (1,2 propanediol). Cows from each block were then randomly allocated to a Latin square and received 6 of the 8 dietary treatments: LF x Urea x Control LF x Urea x 80NOP LF x Nitrate x Control LF x Nitrate x 80NOP HF x Urea x Control HF x Urea x 80NOP HF x Nitrate x Control HF x Nitrate x 80NOP. The experiment ran for 126 days total and consisted of six periods of 21 days each. Each period consisted of an adaptation period of 14 days and a data collection period of 7 days. Cows were provided with partial mixed ration (PMR) twice daily in their own individual feeding troughs at approximately 10:30 and 20:30, allowing for continuous intake throughout the day. The treatment and control compounds were mixed into PMR (specific details not reported).
Study design:	Randomised controlled trial.
Outcome Studied:	Data was collected on rumen gas emissions, milk production, feed consumption, apparent total tract digestibility of nutrients (%), milk composition, body weight, and haematology.
Main Findings (relevant to PICO question):	Cows that received 3-NOP as part of their dietary treatment had decreased milk yield (11.7%) (P < 0.01) than cows receiving the control. This difference was also significantly more pronounced in multiparous cows than primiparous cows (P <0.01). Other outcomes studied that are not relevant to the PICO question will not be discussed further.
Limitations:	The study exhibits selection bias towards a higher producing dairy breed (Holsteins only). The study investigated the effects of 3-NOP on cows past the peak lactation period (> 4–8 weeks postpartum). The study only investigated a single low dose of the compound (80 mg of 3-NOP/kg of feed dry matter), as 60 mg of 3-NOP/kg of feed dry matter is the minimum dose approved by the European Food Safety Authority. The experiment was initially designed as an 8x8 balanced Latin square, but the last 2 periods were discarded such that cows only received 6 of the 8 dietary treatments throughout the study. Possible carryover effects cannot be excluded due to Latin square experimental design and current lack of knowledge on adequate washout periods for 3-NOP. The experimental period was too short in duration (< 28 days) to address whether repeated or long-term use of 3-NOP may result in adaptation by the rumen microbiota. No specific details on how 3-NOP was mixed into the PMR were included in their experimental methods, increasing the possibility of uneven distribution of 3-NOP throughout the PMR. Two of the authors are listed as employees of dsm-firmenich, manufacturer of Bovaer^®^, the tradename for 3-NOP.

**Melgar et al. (2020a):** Effects of 3-nitrooxypropanol on rumen fermentation, lactational performance, and resumption of ovarian cyclicity in dairy cows **Aim: **To determine the effects of 3-nitrooxypropanol (3-NOP) on enteric methane emission, lactational performance, and ovarian cyclicity in early-lactation Holstein cows. The study also examined subjective measures of the sensory properties of the milk.

Population:	Primiparous and multiparous Holstein cows within 3 days after calving, housed in a tie stall barn (Pennsylvania State University's Dairy Teaching and Research Centre, USA).
Sample size:	56 cows.
Intervention details:	Cows were first blocked by parity and calving date. Multiparous cows were further blocked by lactation number and previous lactation milk yield, while primiparous cows were further blocked by predicted milk yield, body weight and body condition score (BCS). This resulted in cows being blocked into 28 blocks, consisting of 2 cows each. Eight of the cows in the experiment were fitted with ruminal cannulas. Cows from each block were randomly allocated to one of two treatment groups: Control: silicon dioxide (SiO_2_) and propylene glycol (1,2 propanediol) only (n = 28) 60NOP: 60 mg of 3-NOP/kg of feed dry matter, mixed with a carrier of SiO_2_ and propylene glycol (1,2 propanediol) (n = 28). The experiment was conducted in phases due to limited tie stall space in experimental barn, each consisting of an experimental period lasting 15 weeks (from 3–105 days in milk (DIM). Cows were provided a total mixed ration (TMR) of feed at approximately 08:00, allowing for continuous intake throughout the day. Feed was further pushed up to the cows 4 to 6 times daily. The treatment and control compounds were mixed into TMR using separate stationary industrial mixers.
Study design:	Randomised controlled trial.
Outcome Studied:	Data was collected on rumen gas emissions, milk production, feed consumption, apparent total tract digestibility of nutrients (%), nitrogen utilisation, rumen digesta and fluid composition, milk composition, body weight, Body Conditions Score (BCS), hormone concentrations, and the resumption of ovarian cyclicity.
Main Findings (relevant to PICO question):	Milk yield did not differ significantly between the control and treatment groups (P = 0.91). Other outcomes studied that are not relevant to the PICO question will not be discussed further.
Limitations:	No power calculation was reported for the experiment. The experiment was non-blinded. The study exhibits selection bias towards a higher producing dairy breed (Holsteins only). Only 8 cows were fitted with ruminal cannulas, thereby receiving a different treatment to the remainder of the study population. It is not clear whether cannulation and/or ruminal sampling has any effect on milk yield and thus cannot be ruled out for this experiment. The study only investigated a single low dose of the compound, as 60 mg of 3-NOP/kg of feed dry matter is the minimum dose approved by the European Food Safety Authority. The lack of a covariate period before each study phase likely prevented the collection of accurate baseline data for milk yield and methane emissions, resulting in the interaction between treatment and study phase (P ≥ 0.06) in their final statistical model. The study failed to quantify the concentration of 3-NOP in the refusals of the TMR, so the exact dosage of 3-NOP that cows received cannot be confirmed. One of the authors is listed as an employee of dsm-firmenich, manufacturer of Bovaer^®^, the tradename for 3-NOP.

**Melgar et al. (2020b):** Dose-response effect of 3-nitrooxypropanol on enteric methane emissions in dairy cows **Aim: **To determine the effect of 3-nitrooxypropanol (3-NOP) on enteric methane emissions, dry matter intake (DMI), and lactational performance of dairy cows when administered in doses of up to 200 mg of 3-NOP/kg of feed dry matter (DM).

Population:	Multiparous Holstein cows housed in a tie stall barn (Pennsylvania State University Dairy Teaching and Research Centre, USA).
Sample size:	49 cows.
Intervention details:	Cows were blocked by days in milk (DIM), enteric methane emissions, and milk yield into 7 blocks, consisting of 7 cows each. Cows from each block were randomly allocated to one of seven treatment groups. The control treatment included silicon dioxide (SiO_2_) and propylene glycol (1,2 propanediol). Six further treatments consisted of SiO_2_, propylene glycol (1,2 propanediol), mixed with a specific volume of 3-NOP/kg of feed dry matter (DM). These were 40 mg, 60 mg, 80 mg, 100 mg, 150 mg, and 200mg. The experiment was conducted in two phases due to limited tie stall space in the experimental barn. Each phase consisted of a covariate period lasting 14 days and an experimental period lasting 17 days. Cows were provided a total mixed ration (TMR) of feed at approximately 08:00 allowing for continuous intake throughout the day. The treatment and control compounds were mixed into the TMR using separate stationary industrial mixers. The 3-NOP diets were prepared in order from lowest to highest concentration.
Study design:	Randomised controlled trial.
Outcome Studied:	Data was collected on rumen gas emissions, milk production, feed consumption, milk composition, and body weight.
Main Findings (relevant to PICO question):	No statistically significant difference in milk yield, but the authors did observe a linear trend for a decrease in milk yield as the 3-NOP dose increased: control vs. all 3-NOP treatments (P = 0.43) linear effect of 3-NOP (P = 0.10) quadratic effect of 3-NOP (P = 0.86). Other outcomes studied that are not relevant to the PICO question will not be discussed further.
Limitations:	No power calculation was reported for the experiment. The experiment was non-blinded. The study exhibits selection bias towards a higher producing dairy breed (Holsteins only). There was also a selection bias towards higher producing multiparous cows, as primiparous cows were not included in this study population. The study investigated the effects of 3-NOP on cows past the peak lactation period (> 4–8 weeks postpartum). The experimental period was too short in duration (< 28 days) to address whether repeated or long-term use of 3-NOP may result in adaptation by the rumen microbiota. One of the authors is listed as an employee of dsm-firmenich, manufacturer of Bovaer^®^, the tradename for 3-NOP.

**Melgar et al. (2021):** Enteric methane emission, milk production, and composition of dairy cows fed 3-nitrooxypropanol **Aim: **To determine the effect of 3-nitrooxypropanol (3-NOP) on enteric methane emissions, as well as milk production and composition in high-yielding dairy cows.

Population:	Primiparous and multiparous Holstein cows housed in a free stall barn (Pennsylvania State University Dairy Teaching and Research Center, USA).
Sample size:	48 cows.
Intervention details:	Cows were initially blocked by lactation number, days in milk (DIM), and enteric methane emissions. Cows were further blocked by current milk yield if multiparous, or genetic potential for milk if primiparous, resulting in 20 blocks consisting of 2 cows each. Cows from each block were randomly allocated to one of two treatment groups: Control: silicon dioxide (SiO_2_) and propylene glycol (1,2 propanediol) only (n = 24) 60NOP: 60 mg of 3-NOP/kg of feed dry matter, mixed with a carrier of SiO_2_ and propylene glycol (1,2 propanediol) (n = 24). The experiment consisted of a covariate period lasting 3 weeks and an experimental period lasting 15 weeks. Cows were provided a total mixed ration (TMR) of feed in the morning allowing for continuous intake throughout the day. The treatment and control compounds were mixed into TMR using separate stationary industrial mixers.
Study design:	Randomised controlled trial.
Outcome Studied:	Data was collected on rumen gas emissions, milk production, feed consumption, milk composition, and body weight.
Main Findings (relevant to PICO question):	Milk yield did not differ significantly between the control and treatment groups (P = 0.74). Four cows were removed from the study, including two due to injury, one due to extremely low dry matter intake (DMI), and another due to failure to visit the GreenFeed® methane measuring system. The data from these cows, along with their blocking pairs, was therefore removed for a total of 8 cows missing from the mixed model. Other outcomes studied that are not relevant to the PICO question will not be discussed further.
Limitations:	No power calculation was reported for the experiment. The experiment was non-blinded. The study exhibits selection bias towards a higher producing dairy breed (Holsteins only). The study investigated the effects of 3-NOP on cows past the peak lactation period (> 4–8 weeks postpartum). The study only investigated a single low dose of the compound, as 60 mg of 3-NOP/kg of feed dry matter is the minimum dose approved by the European Food Safety Authority. The study failed to quantify the concentration of 3-NOP in the refusals of the TMR, so the exact dosage of 3-NOP that cows received cannot be confirmed. Two of the authors are listed as employees of dsm-firmenich, manufacturer of Bovaer^®^, the tradename for 3-NOP.

**Schilde et al. (2021):** Effects of 3-nitrooxypropanol and varying concentrate feed proportions in the ration on methane emission, rumen fermentation and performance of periparturient dairy cows **Aim: **To determine the effects of 3-nitrooxypropanol (3-NOP) in combination with varying proportions of dietary concentrates on methane emissions, rumen fermentation, milk production and energetic efficiency in dairy cows.

Population:	Multiparous Holstein cows (Friedrich-Loeffler-Institut, Brunswick, Germany).
Sample size:	58 cows.
Intervention details:	The groups were balanced for date of calving, fat-corrected milk yield in their previous lactation, Body Condition Score (BCS) and lactation number. 10 cows in the experiment were fitted with ruminal cannulas. Three cannulated cows were allocated to each 3-NOP group and two cannulated cows to each control group. Dietary treatments were organised in a 2 × 2 factorial arrangement with 2 levels of dietary concentrates and 3-nitrooxypropanol (3-NOP) The low concentrate (LC) treatment received a diet consisting of 30% concentrate feed and the high concentrate (HC) treatment received a diet consisting of 55% concentrate feed. The control treatment consisted of only silicon dioxide (SiO_2_) and propylene glycol (1,2 propanediol), while the 60NOP treatment consisted of 60 mg of 3-NOP/kg of feed dry matter, mixed with a carrier of SiO_2_ and propylene glycol (1,2 propanediol). Cows were randomly allocated to receive one of 4 dietary treatments: CON x HC (n = 14) CON x LC (n = 15) 60NOP x HC (n =14) 60NOP x LC (n = 15). The experiment ran for 148 days total (from 28 days ante partum to 120 days postpartum) Cows were provided a PMR of feed consisting of 90% silages, and 10% of a pelleted concentrate containing either 3-NOP for the treatment groups or the placebo for control groups. The partial mixed ration (PMR) was distributed into weighing troughs at 08:00, allowing for continuous intake of the basal diet throughout the day. Concentrate pellets containing 3-NOP or the placebo were supplemented via automatic concentrate feeders for the HC groups (antepartum) as well as all groups (postpartum) to deliver the target 3-NOP concentration.
Study design:	Randomised controlled trial.
Outcome Studied:	Data was collected on rumen gas emissions, milk production, feed consumption, rumen digesta and fluid composition, milk composition, body weight, and Body Conditions Score (BCS).
Main Findings (relevant to PICO question):	The control groups (CON x HC and CON x LC) did not significantly differ in milk yield from the treatment groups (60NOP x HC and 60NOP x LC) (P = 0.270). Three cows from the 60NOP x LC group were removed from the study, including two due to abomasal displacement, and one due to necrotising endometritis. Other outcomes studied that are not relevant to the PICO question will not be discussed further.
Limitations:	No power calculation was reported for the experiment. The experiment was non-blinded. The study exhibits selection bias towards a higher producing dairy breed (Holsteins only). There is also a selection bias towards higher producing multiparous cows, as primiparous cows were not included in this study population. Only 10 cows were fitted with ruminal cannulas, thereby receiving a different treatment to the remainder of the study population. It is not clear whether cannulation and/or ruminal sampling has any effect on milk yield and thus cannot be ruled out for this experiment. The study only investigated a single low dose of the compound, as 60 mg of 3-NOP/kg of feed dry matter is the minimum dose approved by the European Food Safety Authority. No specific details on how 3-NOP was incorporated into the concentrates was included in their experimental methods. The dose of 3-NOP quantified in the PMR was not within acceptable limits (± 10%) of the target dose of 3-NOP.

**Van Gastelen et al. (2020):** 3-Nitrooxypropanol decreases methane emissions and increases hydrogen emissions of early lactation dairy cows, with associated changes in nutrient digestibility and energy metabolism **Aim: **To determine the effects of 3-nitrooxypropanol (3-NOP) on methane and hydrogen emissions, feed intake, energy and nitrogen balance, milk fatty acid profile, milk production, and milk composition in postpartum dairy cows.

Population:	Multiparous Holstein-Friesian cows within 3 days after calving, housed in a tie stall barn (Wageningen University, Netherlands).
Sample size:	16 cows.
Intervention details:	Cows were blocked by calving date, parity, and previous lactation milk yield into 8 blocks, consisting of 2 cows each. Cows from each block were randomly allocated to one of two treatment groups. Control: silicon dioxide (SiO_2_) and propylene glycol (1,2 propanediol) only (n = 8) 60NOP: 60 mg of 3-NOP/kg of feed dry matter, mixed with a carrier of SiO_2_ and propylene glycol (1,2 propanediol) (n = 8). The experiment consisted of a covariate period lasting 3 weeks and an experimental period lasting from 3 days after calving until 115 days in milk (DIM). Cows were provided a total mixed ration (TMR) of feed in the morning allowing for continuous intake throughout the day. The treatment and control compounds were mixed into TMR using an industrial mixer, with 3-NOP mixed after the control.
Study design:	Randomised controlled trial.
Outcome Studied:	Data was collected on rumen gas emissions, milk production, feed consumption, apparent total tract digestibility of nutrients, milk composition, body weight, energy, and nitrogen balance.
Main Findings (relevant to PICO question):	Milk yield did not differ significantly between the control and treatment groups (P = 0.601). One control animal was removed from the experiment due to accidental injury, and single measurements from a control cow and a treatment cow were removed from the analysis due to outlying values for methane emissions and/or milk yield. Other outcomes studied that are not relevant to the PICO question will not be discussed further.
Limitations:	No power calculation reported, so it is unclear whether the limited sample size was adequate to detect differences between treatment and control groups. The experiment was non-blinded. The study exhibits selection bias towards a higher producing dairy breed (Holstein-Friesians only). There was also a selection bias towards higher producing multiparous cows, as primiparous cows were not included in this study population. Separate feed mixers were not used to distribute the 3-NOP and control into the TMR. The study only investigated a single low dose of the compound, as 60 mg of 3-NOP/kg of feed dry matter is the minimum dose approved by the European Food Safety Authority. The study failed to quantify the concentration of 3-NOP in the refusals of the TMR, so the exact dosage of 3-NOP that cows received cannot be confirmed. Two of the authors are listed as employees of dsm-firmenich, manufacturer of Bovaer^®^, the tradename for 3-NOP.

**Van Gastelen et al. (2022):** Methane mitigation potential of 3-nitrooxypropanol in lactating cows is influenced by basal diet composition **Aim: **To determine whether the effect of 3-nitrooxypropanol (3-NOP) on ruminal methane emissions was affected by the basal diet (BD) composition of dairy cows.

Population:	Primiparous and multiparous Holstein-Friesian cows housed in a free stall barn (Wageningen Livestock Research - Dairy Campus, Netherlands).
Sample size:	64 cows.
Intervention details:	Cows were blocked according to parity, milk yield, and days in milk (DIM) into 11 blocks. Ten blocks consisted of 6 cows and 1 block consisted of 4 cows. Cows within each block were then randomly assigned to 1 of 3 diets, and received this from the beginning of the adaptation period to the end of the experiment: A grass-silage (GS) based diet (30% concentrates + 70% grass silage) (n = 22) A grass-silage and corn-silage (GSCS) mixed diet (30% concentrates + 42% grass silage, 28% corn silage) (n = 22) A corn-silage (CS) based diet (30% concentrates + 14% grass silage, 56% corn silage) (n = 20). In the first crossover trial, cows within each basal diet group received either: Control: silicon dioxide (SiO_2_) and propylene glycol (1,2 propanediol) only 60NOP: 60 mg of 3-NOP/kg of feed dry matter (DM), mixed with a carrier of SiO_2_ and propylene glycol (1,2 propanediol). In the second crossover trial, cows within each basal diet group received either: Control: SiO_2_ and propylene glycol (1,2 propanediol) only 80NOP: 80 mg of 3-NOP/kg of feed dry matter (DM), mixed with a carrier of SiO_2_ and propylene glycol (1,2 propanediol). The experiment ran for 70 days total and consisted of two periods of 35 days each. Each period consisted of two adaptation periods of 7 and 14 days and two data collection periods of 7 days. The feed bins were equipped with an automated identification system that used transponders within the collars of the dairy cows to enable access to feed bins throughout the day. Diets were provided as total mixed ration (TMR) in feed bins that recorded feed intake automatically. The treatment and control compounds were mixed into the TMR 3x daily using an automatic feed mixing robot. The control diet containing the placebo was mixed first, followed by a rinsing diet that was not given to the cows. The 3-NOP diets were then mixed, followed again by another rinsing diet.
Study design:	Randomised controlled trial.
Outcome Studied:	Data was collected on rumen gas emissions, milk production, feed consumption, milk composition, and body weight.
Main Findings (relevant to PICO question):	Milk yield did not differ significantly between the control and 60 mg of 3-NOP/kg of feed DM (P = 0.55), but milk yield decreased for 80 mg of 3-NOP/kg of feed DM compared with the control (P < 0.01). One cow was removed from the dataset, due to persistent stealing behaviour and consumption of treatment diets containing 3-NOP doses other than the one assigned. Other outcomes studied that are not relevant to the PICO question will not be discussed further.
Limitations:	No power calculation was reported for the experiment. The experiment was non-blinded. The study exhibits selection bias towards a higher producing dairy breed (Holstein-Friesians only). The study investigated the effects of 3-NOP on cows past the peak lactation period (> 4–8 weeks postpartum). The blocking of the cows cannot be considered complete as one block contained only 4 cows instead of 6. The study had to be completed as 2 crossover trials as it was only possible to mix 2 doses of 3-NOP in the barn at any time. Consequently, 60NOP and 80NOP could never be fed simultaneously. Possible carryover effects cannot be excluded due to cross-over experimental design and current lack of knowledge on adequate washout periods for 3-NOP. The experimental period was too short in duration (< 28 days) to address whether repeated or long-term use of 3-NOP may result in adaptation by the rumen microbiota. Due to the experimental design, the data had to be split into 2 datasets (one for each crossover trial). Statistical comparisons were therefore performed for each 3-NOP dose (60NOP and 80NOP) versus the control separately. Consequently, direct statistical comparisons cannot be made for 60NOP vs. 80NOP. The study only investigated two relatively low doses of the compound, as all doses were less than the maximum concentration of 100 mg of 3-NOP/kg of feed dry matter approved by the European Food Safety Authority. One of the authors is listed as an employee of dsm-firmenich, manufacturer of Bovaer^®^, the tradename for 3-NOP.

**Van Gastelen et al. (2024):** Reducing enteric methane emissions from dairy cattle: Two ways to supplement 3-nitrooxypropanol **Aim: **To determine whether the effects of 3-nitrooxypropanol (3-NOP) on enteric methane emissions and milk production varied if mixed in with roughage or incorporated into a concentrate pellet.

Population:	Holstein-Friesian cows housed in a free stall barn (Flanders Research Institute for Agriculture, Fisheries and Food, Belgium).
Sample size:	30 cows.
Intervention details:	Cows were put into three groups, consisting of 10 cows each, using a balanced randomisation procedure based on days in milk (DIM), parity, milk yield, milk composition, body weight, dry matter intake (DMI) and methane emissions. Each group was randomly assigned to one of three treatments: Control: a mixture of soybean meal and soybean oil in the partial mixed ration (PMR) only (n = 10) 75NOP_(CONC)_: 75 mg of 3-NOP/kg of feed dry matter (administered as 1600 mg of 3-NOP/cow/day) mixed with soybean meal and soybean oil and incorporated into a concentrate pellet (n = 10) 75NOP_(PMR)_: 71.7 mg of 3-NOP/kg of feed dry matter (administered as 1600 mg of 3-NOP/cow/day) mixed with soybean meal and soybean oil and incorporated into PMR (n = 10). The experiment consisted of an adaptation period of 7 days to allow the cows to adapt to their basal diet, a covariate period of 2 weeks when baseline measurements were taken, and an experimental period of 10 weeks. A washout period of 2 weeks was also implemented between experimental periods. Cows had access to feed bins containing partial mixed ration (PMR), and feed stations in and out of the parlour, dispensing concentrates at all times. For the PMR, the 3-NOP mixture (treatment) or soybean meal + soybean oil (control) were mixed into the PMR; specific details not reported. For the concentrates, the 3-NOP mixture (treatment) was mixed with soybean meal + soybean oil and incorporated into pellets using a Promill^®^ press.
Study design:	Randomised controlled trial.
Outcome Studied:	Data was collected on rumen gas emissions, milk production, feed consumption, and milk composition.
Main Findings (relevant to PICO question):	Milk yield did not differ significantly between the control and the 75NOP_(CONC) _group (P = 1.00) or the 75NOP_(PMR) _group (P = 1.00). Data for one cow (control) was completely removed from the study due to lameness. In addition, all data from week 3 (the first week of the experimental period) was removed due to technical complications. Other outcomes studied that are not relevant to the PICO question will not be discussed further.
Limitations:	No power calculation was reported for the experiment. The experiment was non-blinded. The study exhibits selection bias towards a higher producing dairy breed (Holstein-Friesians only). The parity of the cows included in this experiment is not reported. The study investigated the effects of 3-NOP on cows past the peak lactation period (> 4–8 weeks postpartum). The study only investigated a single low dose of the compound (75 mg of 3-NOP/kg of feed dry matter), as 60 mg of 3-NOP/kg of feed dry matter is the minimum dose approved by the European Food Safety Authority. No specific details on how 3-NOP was mixed into the PMR were included in their experimental methods, increasing the possibility of uneven distribution of 3-NOP throughout the PMR. The study failed to quantify the concentration of 3-NOP in the refusals of the PMR, thus the exact dosage of 3-NOP that cows received cannot be confirmed. It is not clear whether cows in the 75NOP_(PMR)_ received any placebo concentrates that did not contain 3-NOP, which may constitute an improper control. It is also not clear whether the 3-NOP compound was in a carrier of silicon dioxide (SiO_2_) and propylene glycol (1,2 propanediol) as with previous experiments, which may also constitute an improper control. Two of the authors are listed as employees of dsm-firmenich, manufacturer of Bovaer^®^, the tradename for 3-NOP.

## Appraisal, application and reflection

In the UK, livestock production is the largest source of methane emissions, with enteric methane accounting for up to 90% of the methane emitted by agriculture (Jebari et al. 2024). Feed additives that inhibit methanogenic archaea within the rumen have therefore emerged as a promising solution to reduce enteric methane emissions in ruminants (Hristov et al. 2013). One such compound is 3-nitrooxypropanol (3-NOP), the active ingredient in Bovaer^®^ (Duval and Kindermann 2012, 2018). Bovaer^®^ is a commercial feed additive produced by dsm-firmenich that has recently been authorised for sale in over 55 countries, including the UK (Weiss 2023) and the EU (Bampidis et al. 2021). 3-nitrooxypropanol functions as a chemical inhibitor of methyl coenzyme M reductase, the enzyme which catalyses the final step of methanogenesis in ruminal archaea (Duin et al. 2016). In dairy cattle, 3-NOP has been reported to effectively reduce methane emissions (g/day) by approximately 30% at an average dose of 70 mg of 3-NOP/kg of feed DM of feed dry matter (DM) (Kebreab et al. 2023). The efficacy of 3-NOP also appears to be more pronounced in dairy cattle than beef cattle (Dijkstra et al*.* 2018), thereby providing a significant opportunity for the dairy industry.

The efficacy of current and future strategies for methane mitigation, however, must be evaluated in the context of One Health. One Health is an integrative framework for addressing global health issues which acknowledges that human, animal, and ecological health are all interdependent (World Organisation for Animal Health, 2024). In the context of dairy production, a “One Health” approach is necessary to find effective solutions to reduce the industry’s environmental impact whilst also safeguarding public health and animal welfare (Nguyen et al., 2022). For example, consequences on milk production must be considered before implementing feed additive programs to ensure that reductions in greenhouse gas emissions are not achieved at the expense of animal welfare or productivity (Llonch et al. 2017). Compounds that negatively affect milk yield or provide no other production benefits are also unlikely to be adopted by dairy producers, regardless of their environmental benefits (Hristov et al. 2013). The partnership between farmers and veterinary surgeons therefore plays a fundamental role in ensuring the future sustainability of dairy production.

Currently, the US patent for 3-NOP claims it is beneficial for overall animal performance, including milk yield (Duval and Kindermann 2018). Two meta-analyses (Jayanegara et al. 2018; Kim et al. 2020) investigating the effects of 3-NOP on sheep, beef and dairy cattle, however, came to conflicting conclusions regarding its effect on milk production. Kim et al. (2020) concluded that 3-NOP tended to decrease milk yield (P = 0.0606), while Jayanegara et al. (2018) concluded that 3-NOP did not have any significant impact on milk yield (P = 0.116). The effect of 3-NOP on milk yield therefore remains unclear. This Knowledge Summary therefore aims to evaluate whether milk yield may be affected in dairy cows receiving the methanogenesis inhibitor 3-NOP (Bovaer^®^), compared to those that receive no intervention to reduce enteric methane emissions.   

The primary objective of all critically analysed studies, however, was to determine the efficacy and persistency of 3-NOP in reducing methane emissions, with milk yield only serving as a secondary objective. While all the experiments collected data on milk production, no study specifically sought to solely address the PICO question. Furthermore, three studies aimed to investigate the combined effects of 3-NOP with diet composition (Kjeldsen et al., 2024; Van Gastelen et al., 2022; Schilde et al., 2021) or other methanogenesis inhibitors (Maigaard et al., 2024; Ma et al., 2024), which further limits the strength of the evidence provided by the current research. Finally, in the study conducted by Lopes et al. (2016), the effects of 3-NOP were likely confounded by the administration of recombinant bovine somatotropin (bST) as this may have increased milk yield (Fluck et al., 2024) and therefore masked the effect of 3-NOP on milk production.

All studies featured either Holstein and/or Holstein-Friesian cows, while only one study featured Brown Swiss cows (Ma et al., 2024). Although milk yield was not affected by treatment of 3-NOP for either breed, there were other unexpected breed effects (Ma et al., 2024).  For example, 3-NOP had a reduced methane mitigation effect on Brown Swiss cows and only reduced methane for approximately 4 hours after feeding, while effects persisted for 10 hours in Holstein-Friesian cows (Ma et al., 2024). The results of these studies can therefore not be reliably generalised to all dairy herds, as the effects of 3-NOP on milk yield could be more pronounced in lower-producing pedigree breeds or other crossbreds. Consequently, the reviewed studies only provide moderate evidence to answer the PICO question, and future studies should aim to quantify the effects of 3-NOP in additional dairy breeds.

Nine of the reviewed studies included both primiparous and multiparous cows in their study population (Haisan et al., 2014; 2017; Lopes et al., 2016; Hristov et al., 2015; Melgar et al., 2020a; 2021; Van Gastelen et al., 2022; 2024; Maigaard et al., 2024). Five of the studies, however, exhibit a selection bias towards multiparous cows, as primiparous cows were not included in these experiments (Melgar et al., 2020b; Van Gastelen et al., 2020; Schilde et al., 2021; Ma et al., 2024; Kjeldsen et al., 2024). Total milk production tends to increase with lactation number (Hansen et al., 2006; Masía et al., 2020; Marumo et al., 2022; Melgar et al., 2020a) due to differences in energy partitioning between primiparous and multiparous cows during early lactation (Wathes et al., 2007). Primiparous animals must continue to partition nutrients towards tissue synthesis as growth is still an energetic priority, ultimately limiting the bodily resources available for milk production during their first lactation (Wathes et al., 2007). The effects of 3-NOP on milk yield could therefore be more pronounced in lower-producing primiparous cows. Thus, by excluding primiparous cows, the results may become biased by higher-producing multiparous cows. Studies which included cows of all parities provide stronger evidence to answer the PICO question (Haisan et al., 2014, 2017; Lopes et al., 2016; Hristov et al., 2015; Melgar et al., 2020a, 2021; Van Gastelen et al., 2022, 2024; Maigaard et al., 2024).

Most of the studies failed to account for the effects of 3-NOP on milk yield throughout the different stages of lactation. Eleven of the reviewed studies only investigated the effects of 3-NOP on cows in mid- to late lactation, after the cows had passed the peak lactation period (Haisan et al., 2014, 2017; Hristov et al., 2015; Lopes et al., 2016; Van Wesemael et al., 2019; Melgar et al., 2020b, 2021; Van Gastelen et al., 2022; Maigaard et al., 2024; Ma et al., 2024; Kjeldsen et al., 2024). In contrast, Melgar et al. (2020a) and Van Gastelen et al. (2020) examined the effects of 3-NOP throughout the peak lactation period, from 3 days after calving up to 105 and 115 days in milk (DIM), respectively. Schilde et al. (2021) also included the prelactation period by examining the effects of 3-NOP from 28 days antepartum to 120 days postpartum. Van Gastelen et al. (2024), however, was the only study to observe the effects of 3-NOP for an entire year, across all stages of lactation. As lactation is a dynamic process, it is important to investigate the effects of 3-NOP throughout different stages of milk production. Peak lactation is a critical period to examine the effects of 3-NOP, as early-lactation cows undergo a series of metabolic changes to meet the energetic demands of peak milk production and, as a result, lose body condition and enter a state of negative energy balance (Kirkland & Gordon, 2001; De Vries & Veerkamp, 2000). Consequently, any effects of 3-NOP on milk yield may be more pronounced during the peak lactation period, between 4 and 8 weeks after calving (Silvestre et al., 2009). Results derived from cows past the peak lactation period may therefore not be indicative of the effect of 3-NOP across various stages of lactation. Studies which span multiple stages of lactation, including the peak lactation period (Melgar et al., 2020a; Van Gastelen et al., 2020, 2024; Schilde et al., 2021),  provide stronger evidence to answer the PICO question.

Significant limitations in the experimental design of the critically reviewed studies include the lack of power analyses, blinding, and inadequate detail of the randomisation techniques used. Multiple studies (Haisan et al., 2014, 2017; Lopes et al., 2016; Kjeldsen et al., 2024; Van Gastelen et al., 2020; Ma et al., 2024) did not report a power analysis while using small sample sizes (less than 16 cows) in their experiments. In several instances, this was likely to minimise the number of cows with ruminal cannulas necessary for the investigation (Haisan et al., 2014, 2017; Lopes et al., 2016; Kjeldsen et al., 2024). However, of all the studies reviewed, only Van Gastelen et al. (2024) reported a power analysis when determining sample size. With no power analyses reported for the remainder of the studies, it is unclear whether sample sizes were adequate to detect differences between treatment groups/periods, thus reducing the reliability of the results. Furthermore, while all but four studies (Haisan et al., 2014, 2017; Lopes et al., 2016; Kjeldsen et al., 2024) state that cows were randomly assigned to treatments groups or sequences, full details of the method used to generate the random allocation are rarely provided (Petrie & Watson, 2013). Most importantly, all the reviewed studies failed to specify whether animal care staff, the researchers conducting the experiment, or those collecting and analysing the data, were blinded to treatment and control allocation. Randomisation alone is not sufficient to minimise bias and there is significant evidence to support that lack of blinding in animal studies is associated with exaggerated effect sizes (Krauth et al., 2013; Bello et al., 2014). The lack of blinding in the critically reviewed studies undermines the validity of their results and their ability to provide strong evidence to answer the PICO question.

Seven of the reviewed studies utilised a Latin square (Haisan et al., 2017; Maigaard et al., 2024; Ma et al., 2024; Kjeldsen et al., 2024) or cross-over experimental design (Haisan et al., 2014; Lopes et al., 2016; Van Gastelen et al., 2022).  While these experimental designs limit within-animal variability and may require fewer animals than other experimental designs, they can be liable to carry over effects (Mills et al., 2009). Carry-over effects can alter estimates of treatment effects but can be minimised by including adequate “wash-out” periods between treatments to ensure the previous treatment has been completely metabolised (Mills et al., 2009). Although each study included a washout/adaptation period of 7–21 days, it remains unclear if this duration provides an adequate "washout," as there is currently limited research on the absorption, distribution, metabolism, and excretion (ADME) of 3-NOP in dairy cows (Bampidis et al., 2021). Several studies (Melgar et al., 2020b; Reynolds et al., 2014; Van Wesemael et al., 2019) suggest that 3-NOP effect on methane reduction is transient, as enteric emissions of methane return to basal levels within a week of stopping 3-NOP. It is, however, not clear whether 3-NOP could take longer to affect milk yield than the time needed to alter methane emissions. Consequently, possible carryover effects cannot be excluded in these experiments.

The remaining studies used randomised block (Hristov et al., 2015; Melgar et al., 2020a, 2020b, 2021; Van Gastelen et al., 2020, 2024; Van Wesemael et al., 2019) or factorial (Schilde et al., 2021) experimental designs that allowed cows to be blocked according to characteristics such as parity, body weight, or current milk yield. While most studies implemented a 14 to 21-day covariate period to collect baseline data (Hristov et al., 2015; Melgar et al., 2020b, 2021; Van Wesemael et al., 2019; Van Gastelen et al., 2024, 2020), some did not include covariate periods (Schilde et al., 2021; Melgar et al., 2020a). For instance, Melgar et al. (2020a) completed their experiment in two phases due to a limited number of tie-stalls in the experimental barn. The authors, however, do not mention a covariate period and provide no details regarding how the remaining cows were housed during the respective phases. The lack of a covariate period before each study phase likely prevented the collection of accurate baseline data for milk yield and methane emissions. This may also partially explain why an interaction between treatment and study phase (P ≥ 0.06) was identified in their final statistical model (Melgar et al., 2020a). Moreover, as housing systems (tie-stalls versus other types of loose housing) have been demonstrated to have variable effects on milk yield (Beaver et al., 2021), housing conditions should be reported to assist the interpretation of experimental results (Krauth et al., 2013). In contrast, both Hristov et al. (2015) and Melgar et al. (2020b) conducted their experiments in two phases but included a 14-day covariate period for each phase to collect baseline data. In these studies, the inclusion of a covariate period allowed cows to be blocked by their current milk yield and allowed milk yield to be included as a time-dependent covariate in the final model. Consequently, the interaction between treatment and study phase was nonsignificant (P = 0.32) (Melgar et al., 2020b). Studies with covariate periods, therefore, provide stronger evidence to answer the PICO question.

The studies reviewed also had experimental periods of varying duration, including eight short-term studies (less than 28 days) (Haisan et al., 2014, 2017; Lopes et al., 2016; Melgar et al., 2020b; Maigaard et al., 2024; Ma et al., 2024; Kjeldsen et al., 2024; Van Gastelen et al., 2022) and seven longer-term studies (Hristov et al., 2015; Van Wesemael et al., 2019; Melgar et al., 2020a; Van Gastelen et al., 2024, 2020; Melgar et al., 2021; Schilde et al., 2021). Only Van Gastelen et al. (2024) conducted an experimental period that encompassed all stages of lactation, including the dry period. However, this study did not investigate the effect of 3-NOP on the overall shape of the lactation curve. As total milk yield depends on the overall shape of the lactation curve, including peak yield and lactation persistency (Togashi & Lin, 2003), long-term studies are necessary to describe effect of 3-NOP on milk production throughout lactation. Furthermore, most of the studies were too short in duration to address whether repeated or long-term use of 3-NOP may result in adaptation by the rumen microbiota (Hristov et al., 2013; Van Gastelen et al., 2024). Thus, additional studies are necessary to determine the influence of 3-NOP on the overall shape of the lactation curve, including peak yield and lactation persistency, as well as the potential for rumen adaptation.

Current research has primarily investigated the effects of 3-NOP on milk yield at relatively low doses. Six of the studies only assessed a single dose of 60 mg of 3-nitrooxypropanol/kg of feed dry matter (DM) (Lopes et al., 2016; Melgar et al., 2020a, 2021; Van Gastelen et al., 2020; Schilde et al., 2021; Ma et al., 2024), the minimal level recommended by the European Food Safety Authority (EFSA) (Bampidis et al., 2021). As 3-NOP was only evaluated at the minimum concentration, these studies cannot be used to support the effects of 3-NOP at higher doses (Bampidis et al., 2021). Of the four studies that tested a slightly higher dose of 80 mg of 3-nitrooxypropanol/kg of feed DM, Maigaard et al. (2024) and Van Gastelen et al. (2022) both observed a significant decrease in milk yield (P < 0.01) in cows fed 3-NOP, while Kjeldsen et al. (2024) found a tendency (P= 0.06) for milk yield to decrease. In contrast, however, Haisan et al. (2014) and Haisan et al. (2017) both tested high doses of 3-NOP (~130 mg of 3-nitrooxypropanol/kg of feed DM) and found no significant difference in milk yield. As a relationship between 3-NOP dosage and milk yield has not yet been described, multiple doses are needed to conduct any form of dose-response analysis. While Hristov et al. (2015) tested three increasing doses of 3-NOP, only Melgar et al. (2020b) studied six doses, including and exceeding the maximum concentration of 100 mg of 3-nitrooxypropanol/kg of feed DM approved by the EFSA (Bampidis et al., 2021). Consequently, Melgar et al. (2020b) was able to observe a trend for milk yield to linearly decrease with increasing 3-NOP dose. These findings suggest that milk yield may be adversely affected by 3-NOP at higher doses. Further research is necessary to determine the effects of 3-NOP at concentrations exceeding 80 mg of 3-nitrooxypropanol/kg of feed DM and an appropriate dose-response model.

Among the reviewed studies, there was variation in the methods used to mix 3-NOP throughout the cows’ total mixed ration (TMR) or partial mixed ration (PMR). Haisan et al. (2014) and Haisan et al. (2017) both manually mixed both the 3-NOP and the control silicon dioxide (SiO_2_) powders into the cows' daily rations, increasing the possibility of uneven distribution of 3-NOP throughout the TMR. It is therefore plausible that cows may have selectively fed to avoid 3-NOP, reducing their intake of the compound.  While several studies used separate industrial mixers for control and treatment groups (Melgar et al., 2020a, 2020b, 2021), others used the same industrial mixer or automatic feed mixing robot for the control diet, followed by the treatment diet (Van Gastelen et al., 2020, 2022, 2024; Kjeldsen et al., 2024). In contrast, Schilde et al. (2021) and Van Wesemael et al. (2019) incorporated the 3-NOP and control mixtures into concentrate pellets. Five studies however, provided no specific details on how 3-NOP was mixed into the cow’s daily ration in their experimental methods (Hristov et al., 2015; Lopes et al., 2016; Van Wesemael et al., 2019; Maigaard et al., 2024; Ma et al., 2024). Nine of the reviewed studies also failed to quantify the concentration of 3-NOP distributed throughout the TMR, despite samples from refusals being collected daily throughout many of the experiments (Haisan et al., 2014, 2017; Hristov et al., 2015; Lopes et al., 2016; Van Wesemael et al., 2019; Melgar et al., 2020a, 2021; Van Gastelen et al., 2020; Ma et al., 2024). As these studies did not quantify the concentration of 3-NOP in refusals, it is not certain how uniformly dispersed the compound was within the TMR, and thus the exact dosage of 3-NOP that cows received cannot be confirmed. Of the studies that did quantify the concentration of 3-NOP in feed refusals (Melgar et al., 2020b; Schilde et al., 2021; Van Gastelen et al., 2022, 2024; Maigaard et al., 2024; Kjeldsen et al., 2024), only four determined the administered doses to be within acceptable limits (± 10% of the target doses of 3-NOP). In contrast, both Schilde et al. (2021) and Van Gastelen et al. (2024) both found that on average cows received significantly less than the intended doses of 60 mg of 3-nitrooxypropanol/kg and 80 mg of 3-nitrooxypropanol/kg of feed DM, respectively. Thus, in most of the reviewed studies, the amount of 3-NOP that cows received was either unknown or lower than the intended dose. Consequently, most studies are unable to draw conclusions regarding 3-NOP doses greater than 80 mg of 3-nitrooxypropanol/kg of feed DM and only provide moderate evidence to answer the PICO question.

Several studies removed a small number of cows from their experiments due to accidental injury or illnesses such as lameness, abomasal displacement, endometritis, failure to get pregnant, stealing behaviour, missing methane measurements, or outlying values for dry matter intake (DMI) and/or milk yield (Lopes et al., 2016; Haisan et al., 2017; Van Gastelen et al., 2020, 2022, 2024; Melgar et al., 2021; Schilde et al., 2021). Two of the critically reviewed studies, however, differed significantly in their approach to the removal of subjects and subsequent missing data. Both Melgar et al. (2021) and Van Gastelen et al. (2024) removed four animals, along with their blocking pairs, from their experiments at various stages. Melgar et al. (2021) provided justification for the cows’ removal from the experiment, including lack of methane measurements, low DMI or injury. Van Gastelen et al. (2024), however, merely state that the four animals were removed due to health conditions unrelated to 3-NOP or the experiment. Justification for removal, including the details of illness/injury, should be comprehensively reported, as without further detail or investigation it is not certain that the illness or injury is unrelated to the administration of 3-NOP or the experimental conditions. Several studies also differed in their statistical approach to the missing data. Van Gastelen et al. (2024) opted to retain early data from removed cows in the final data set, up until the first sign of illness was observed. This strategy avoided the unnecessary removal of data and allowed observations from the first 25 ± 2.8 weeks of the experimental period to be included in the final data set for these cows (Van Gastelen et al., 2024). Melgar et al. (2021) and Van Wesemael et al. (2019), however, excluded data from removed cows in their final statistical models. This method presents a limitation, as excluding entire subjects can introduce exclusion bias if the data is not missing completely at random (Petrie & Watson, 2013). Consequently, this approach is only suitable when there are relatively few missing values in the dataset, which weakens the overall strength of evidence from these studies.

## Methodology

**Search Strategy:** 

Databases searched and dates covered:	CAB Abstracts (CABI Digital Library) 2000–September 2024 Web of Science (Clarivate Analytics) 2000–September 2024 ScienceDirect (Elsevier) 2000–September 2024
Search strategy:	CAB Abstracts: (3-nitrooxypropanol OR Bovaer) AND (cow OR cattle OR bovine OR bovid*) AND (dairy OR milk OR lactation) AND (yield OR production) Web of Science: (3-nitrooxypropanol OR Bovaer) AND (cow OR cattle OR bovine OR bovid*) AND (dairy OR milk OR lactation) AND (yield OR production) ScienceDirect: (3-nitrooxypropanol OR Bovaer) AND (cow OR cattle OR bovine OR bovid*) AND (dairy OR milk OR lactation) AND (yield OR production)
Dates searches performed:	10 Sep 2024

**Exclusion / Inclusion Criteria:** 

Exclusion:	Did not answer PICO question: Study conducted on other ruminants Administration of other feed additives/ methanogenesis inhibitors Administration of 3-NOP via method other than mixed feed ration or concentrate pellets (e.g. via ruminal cannula) No measurement of milk yield (kg/day). Non-English language. In vitro study. Non-primary research.
Inclusion:	Answers PICO question: Study conducted in dairy cattle. Administration of 3-NOP via mixed feed ration or concentrate pellets. Measurement of milk yield (kg/ day).

**Search Outcome:** 

Database	Number of results	Excluded – did not answer the PICO question	Excluded – non-English language	Excluded – in-vitro study	Excluded – non-primary research	Total relevant papers
CAB Abstracts	46	22	0	4	7	13
Web of Science	88	55	0	8	10	15
Science Direct	179	87	0	5	75	12
Total relevant papers when duplicates removed						15

## Acknowledgements

The author would like to thank Professor Renato Previdelli for their guidance and feedback.

## ORCiD

Justine Cole: https://orcid.org/0000-0003-1516-8618

## Conflict of Interest

The author declares no conflicts of interest.
